# Effectiveness of Natalizumab in Achieving No Evidence of Disease Activity (NEDA-3)—Data From a Local Norwegian Cohort

**DOI:** 10.3389/fneur.2021.765837

**Published:** 2021-10-20

**Authors:** Andreas K. Jaklin, Espen Benjaminsen, Karl B. Alstadhaug

**Affiliations:** ^1^Department of Medicine, University Hospital of North Norway, Tromsø, Norway; ^2^Department of Neurology, Nordland Hospital Trust, Bodø, Norway; ^3^Institute of Clinical Medicine, The Arctic University of Norway, Tromsø, Norway

**Keywords:** multiple sclerosis, natalizumab, NEDA, JCV, effectiveness, survival analysis

## Abstract

**Objective:** We aimed to determine the effectiveness of natalizumab (NTZ) by assessing overall No Evidence of Disease Activity 3 (NEDA-3) in a local Norwegian cohort.

**Background:** NTZ is an immunomodulating drug used in the treatment of multiple sclerosis (MS). It has typically been used as a second-line treatment, but certain patients with high disease activity have started directly with NTZ.

**Methods:** This retrospective cohort study includes all patients who received NTZ for relapsing–remitting MS at Nordland Hospital in the period 2008–2018. In June 2019, status for every patient was assessed, and a survival curve was used to show the cumulative probability of achieving NEDA-3 over time.

**Results:** The cohort consisted of 66 patients, 49 women and 17 men with a mean age of 40.0 ± 10.8 years. Each patient received on average 45.8 ± 36.4 NTZ infusions. Mean age and Expanded Disability Status Scale (EDSS) at first infusion was 34.8 ± 10.5 and 3.2 ± 1.9, respectively. Prior to NTZ treatment, 83% had used other disease modulating drugs and 65% were anti-JC virus (JCV) seronegative. During the study period, seven patients converted to seropositive. In 2019, 40 patients had switched or stopped treatment: 19 due to positive JCV serostatus, 9 due to disease activity, 7 due to adverse effects or complications (1 progressive multifocal leukoencephalopathy), 2 due to pregnancy, and 3 due to autologous hematopoietic cell transplantation abroad. Three patients experienced rebound in the wake of discontinuation (7.5%). Of the patients receiving NTZ for more than 3 years (*n* = 33), 50% had achieved NEDA-3 after 3 years. Compared to those with evidence of disease activity (EDA), these NEDA-3 patients had significant lower EDSS score before first NTZ treatment (*p* = 0.04). They were also slightly, but not significantly, younger at debut of their MS, at the diagnosis and at first NTZ treatment. Of all the patients who ever started on NTZ, 23% had achieved NEDA-3 5 years later. The mean EDSS in 2019 was 3.6 ± 2.5.

**Conclusion:** Despite the high rate of treatment switch, mainly due to the risk of PML, almost one in four who started on NTZ achieved NEDA-3 after 5 years, and the overall disease progression was low in the total cohort. Treating less advanced disease seems to predict better long-term stability.

## Introduction

Multiple sclerosis (MS) is a chronic disease characterized by inflammatory lesions in the central nervous system (CNS). It is regarded as the most common cause of neurologic deficits in young adults (1). A fluctuating process of inflammation, typically seen early in the disease, clinically manifested with transient episodes of neurological symptoms (relapsing–remitting multiple sclerosis, RRMS), affects the majority. As the disease progresses over time, CNS lesions are accumulating, neurodegeneration becomes more evident, and patients progress to a higher disability (2). Traditionally, the Expanded Disability Status Scale (EDSS) (3) has been used to assess disease progression.

Natalizumab (NTZ) is a monoclonal antibody approved as a treatment for RRMS (4). Due to safety concerns, particularly an increased risk of progressive multifocal leukoencephalopathy (PML), treatment has generally been reserved for patients with highly active disease. Most often, NTZ has been given if initial therapy was considered ineffective or poorly tolerated, but sometimes also as initial therapy in aggressive disease. Recently, Beslutningsforum (a decision forum established by The Norwegian Ministry of Health and Care Services) decided that NTZ treatment cannot be initiated in new patients after December 1, 2019.

In the Tysabri Observational Program (TOP), 86.5% of patients, with a median EDSS score of 3.5, did not experience EDSS increases ≥ 1.0 after 24 months of NTZ treatment (5). Besides the TOP data, which are based on active registration and patient consent, relatively few studies have described complete cohorts with real-world data. In recent years, No Evidence of Disease Activity (NEDA) has been used as a surrogate outcome measure in clinical treatment trials (6). We set out to describe all patients treated with NTZ over a 10-year period at a single center, and assess NEDA-3 over time.

## Method

### Patients

All patients with relapsing–remitting MS diagnosed in accordance with the McDonald criteria (7), receiving at least one dose of NTZ at Nordland hospital in the period 2008–2018 were included in this study. The patients received intravenous infusions with 300 mg NTZ every 4 weeks. None of the patients were on extended interval dosing regimen. Side effects were reported to specialized nurses at every infusion and at every outpatient visit (every 6–12 months) carried out by a neurologist.

### Time and Location

This study took place at Nordland Hospital localized in the northern part of Norway. On January 1, 2017, there were 657 patients living with MS out of a population of 242,866 in Nordland (7). All patients with MS treated with NTZ at the hospital, from the first patient in 2008 until June 2018, were included. A final status assessment was made in June 2019.

### Data Acquisition

The patients were identified and scrutinized through an electronic medical record system (DIPS) at the hospital. All patients had been examined by a neurologist or a doctor in training for becoming a neurologist 3 months after the first NTZ infusion, and later every 6–12 months. MRI scans were carried out prior to the patient's appointments. EDSS score was calculated retrospectively by the investigators when missing in the medical recordings. If patients had moved during the follow-up period and treated elsewhere, medical records from their current hospital were obtained. The variables recorded for every study patient are listed in Tables 1–**4**.

**Table 1:** Demographic and diagnostic data for the MS population.

	**Women (*n* = 49)**	**Men (*n* = 17)**	**Total (*n* = 66)**	***p*-value**
**Debut**				
Age at MS-debut (age), mean ± SD	27.4 ± 9.4	25.7 ± 10.7	26.9 ± 9.7	0.53
Debut compatible with posterior myelitis, *n* (%)	19 (39)	6 (35)	25 (38)	0.83
Debut compatible with optic neuritis, *n* (%)	12 (24)	5 (29)	17 (26)	0.79
**Diagnosis**				
Age at diagnosis (years), mean ± SD	29.2 ± 9.1	27.9 ± 9.9	28.9 ± 9.3	0.63
EDSS at time of diagnosis, mean ± SD	2.3 ±1.8	2.1 ± 1.1	2.2 ± 1.6	0.71
Leukocytes in CSF at first examination (*n*), mean ± SD	10.8 ± 9.5	11.3 ± 12.3	1.0 ± 10.2	0.89
Total protein in CSF at first examination (*n*), mean ± SD	0.39 ± 0.14	0.51 ± 0.14	0.42 ± 0.15	0.08
IgG Index in CSF at first examination, mean ± SD	1.27 ± 0.61	1.18 ± 0.62	1.25 ± 0.61	0.68
Time from diagnosis to first treatment (years), mean ± SD	2.1 ± 4.1	0.7 ± 1.7	1.8 ± 3.6	0.05

*CSF, cerebrospinal fluid; EDSS, Expanded Disability Status Scale*.

### Primary Outcome

NEDA-3, a well-accepted outcome measure to assess disability and prognosis in RRMS (8), was the primary outcome of the study. A status of NEDA-3 is based on (1) absence of clinical MS relapse, (2) absence of disability worsening corresponding to ≥1 point increase in EDSS sustained for 6 months, and (3) absence of gadolinium-enhancing lesions or new or enlarged T2-hyperintense lesions on magnetic resonance imaging (MRI) (9).

### Secondary Outcomes

Secondary outcomes included adverse effects of NTZ and causes of drug abruption and switch of treatment. We also searched for differences between those with evidence of disease activity (EDA) and those who achieved NEDA-3.

### Statistical Analysis

A survival curve was used to demonstrate the cumulative probability of having NEDA-3 status over time. The Kaplan–Meier survival method was used to compare the survival curves for males and females. Student's *t*-test was applied to compare demographic data. The threshold for statistical significance was *p* < 0.05. Secondary outcomes are descriptively presented. IBM SPSS statistics version 26 was used for all the analyses.

### Study Approval

The study was approved by the institutional data protection official (PVO 03-18).

## Results

### Demographics and Diagnostic Data

A total of 66 patients with RRMS were treated at Nordland hospital with at least one dose of NTZ in the given study period (Table 1). The cohort consisted of 49 women and 17 men with mean age of 40.0 ± 10.8 years as of 2019. The mean age at diagnosis was 28.9 ± 9.3 years. The mean EDSS score at the time of diagnosis was 2.2 ±1.6. It took on average 1.8 ± 3.6 years from the first symptom of MS until diagnosis.

### Treatment With NTZ

Mean age and EDSS of the patients at first infusion were 34.8 ±10.5 years and 3.2 ± 1.9, respectively (Table 2).

**Table 2:** Treatment with natalizumab.

	**Women (*n* = 49)**	**Men (*n* = 17)**	**Total (*n* = 66)**	***p*-value**
**Before treatment**				
Positive JCV serostatus, *n* (%)	18 (37)	5 (29)	23 (35)	0.79
Patients who have used DMTs, *n* (%)	42 (86)	13 (76)	55 (83)	0.38
Drugs (*n*), mean ± SD	1.2 ± 0.8	1.2 ± 1.0	1.2 ± 0.8	0.95
^*^Duration of prior DMTs (years), mean ± SD	3.4 ± 3.4	3.4 ± 3.3	3.4 ± 3.4	0.96
Clinical attacks last 12 months, mean ± SD	1.38 ± 1.0	1.24 ± 0.8	1.34 ± 1.0	0.62
EDSS, mean ± SD	3.3 ± 2.0	2.9 ± 1.5	3.2 ± 1.9	0.43
EDSS <3.0, *n* (%)	19 (39)	9 (53)	28 (42)	0.31
**Treatment**				
Age (years) at first dose, mean ± SD	32.8 ± 10.7	35.5 ± 10.5	34.8 ± 10.5	0.37
Infusions (*n*), mean ± SD	42.5 ± 33.8	55.3 ± 43.0	45.8 ± 36.4	0.21
Antibodies against natalizumab, *n*	1	0	1	0.55
Leukocytosis, *n* (%)	19 (39)	8 (47)	27 (41)	0.55
JCV-conversion, *n* (%)	5 (10)	2 (12)	7(11)	0.86
^**^Reported side-effects, *n* (%)	14 (29)	2 (12)	16 (24)	0.16
^***^NEDA-3 after 1 year, *n* (%)	37 (76)	12 (71)	49 (74)	0.55
^***^NEDA-3 after 2 years, *n* (%)	33 (67)	12 (71)	45 (68)	0.35
^***^NEDA-3 after 3 years, *n* (%)	21 (43)	11 (65)	33 (50)	0.20
^***^NEDA-3 after 5 years, *n* (%)	10 (20)	5 (29)	15 (23)	0.68
**Drug abruption and switch of treatment**				
Discontinuation due to disease activity, *n* (%)	8 (16)	1 (6)	9 (14)	0.28
Discontinuation due to JCV-status, *n* (%)	15 (30)	4 (24)	19 (29)	0.58
Discontinuation due to other causes^****^, *n* (%)	10 (20)	2 (12)	12 (18)	0.43
Rebound, *n* (%)	2 (4)	1 (6)	3 (5)	0.76

*EDSS, Expanded Disability Status Scale; NA, Not applicable; JCV, JC polyomavirus; NEDA-3, No Evidence of Disease Activity, DMD, Diseasemodulating treatment*.

* *Range 13*

** *Most side effects were mild, but six patients discontinued NTZ because of side effects; one because of hypersensitivity reaction and one due to progressive multifocal leukoencephalopathy (PML)*.

*** *Number of patients who were treated for the given years is in parentheses*.

**** *Pregnancy, adverse effects, and self-financed autologous hematopoietic stem cell transplantation*.

Prior to treatment, 43 (65%) were anti-JC virus (JCV) seronegative, but 7 of these seroconverted to positive during the study. On average, each patient received 45.8 ± 36.4 doses of NTZ (range 137). Eleven patients (17%) started with NTZ as a first-line therapy, and 13 (20%) of the patients started with the treatment within 1 year of the diagnosis. On average, however, it took 7.9 ± 6.5 years after first symptom and 5.9 ± 5.8 years after the diagnosis before treatment with NTZ was given. Most of the patients (55, 83%) had received other disease-modulating therapies (DMTs) prior to NTZ.

The risk of PML was the main reason for NTZ discontinuation. Twenty patients changed treatment to fingolimod, four to alemtuzumab, two to dimethyl fumarate, five to rituximab, and three patients received autologous hematopoietic cell transplantation (aHSCT) abroad. The patients who received aHSCT actually had a stable disease, but still wanted the treatment. They did it at own costs. Five patients did not receive any further treatment, and one patient died with secondary progressive MS. Three patients experienced rebound subsequent to discontinuation of NTZ.

### Survival Analyses

**Figure 1:**
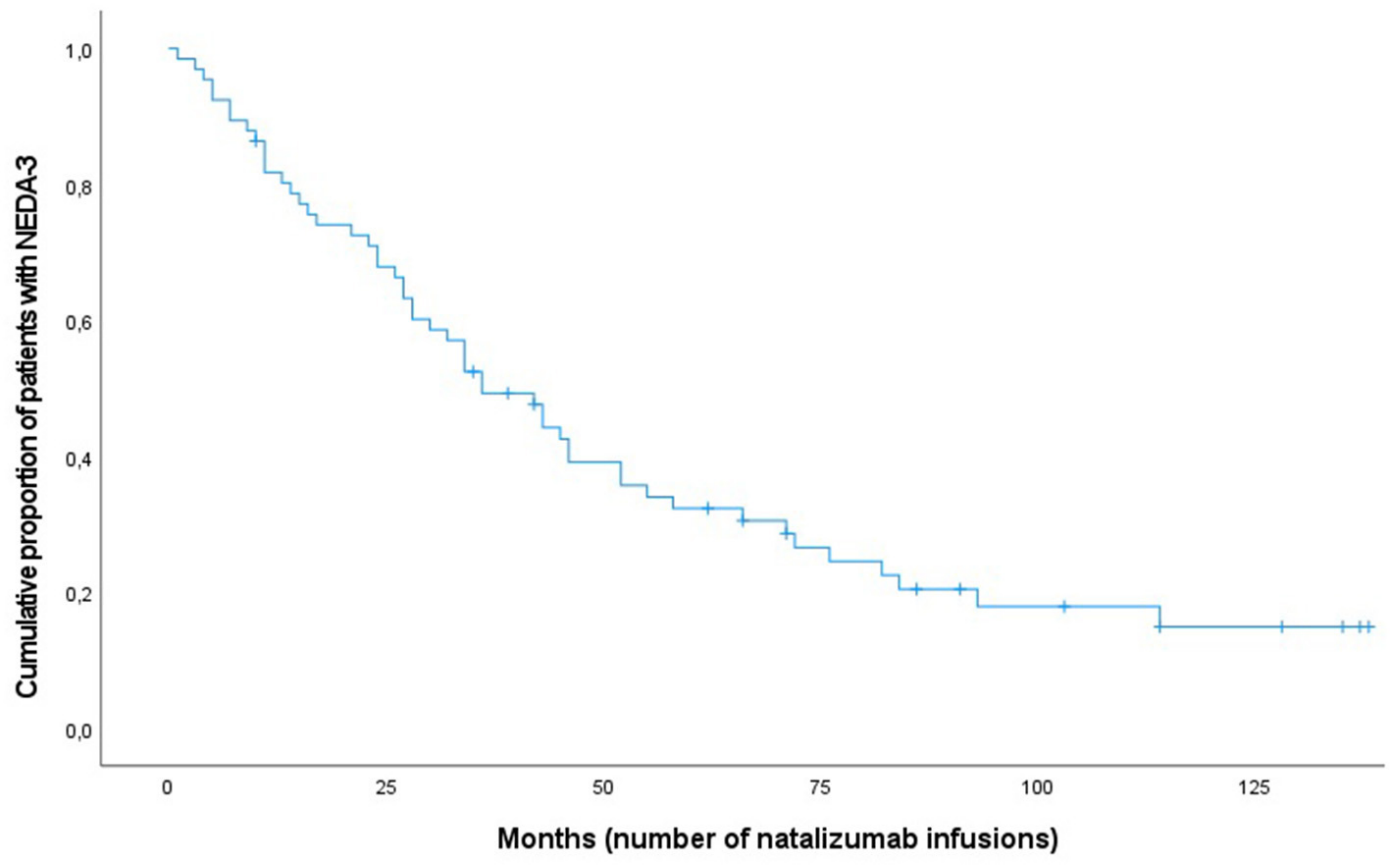
Kaplan-Meier curve for patients with multiple sclerosis (*n* = 66) achieving NEDA-3 following natalizumab treatment.

The Kaplan–Meier survival analysis was used to look at disease activity rates over the study period and to compare the differences in the proportions in males and females. On average, females received 50 infusions before disease activity compared with 63 in males. No significant statistical difference was found (Breslow, *p* = 0.50). Figure 1 shows the cumulative risk over time of disease activity after having started with NTZ treatment.

### Side Effects and Tolerability of NTZ

Side effects were recorded in 16 patients (24%). These included fatigue (*n* = 5), headache (*n* = 2), fever (*n* = 1), hair loss (*n* = 1), hypertension (*n* = 1), influenza-like symptoms (*n* = 1), muscle cramps (*n* = 1), increased infection tendency (*n* = 1), hypersensitivity reaction (*n* = 1), myalgia (*n* = 1), and allergy (*n* = 1). One patient developed urticarial rash during an infusion. Another patient experienced a hypersensitivity reaction during the third infusion, requiring adrenaline. One patient developed PML, but recovered with only minor sequela. An undernourished patient with advanced MS died in secondary progressive disease due to underlying ventricular cancer. Leucocytosis was observed in 41% of the patients.

### Status in 2019

By June 2019, the patients had an average EDSS score of 3.6 ± 2.5. A total of eight patients had progressed to secondary progressive multiple sclerosis (SPMS), all of them women. Thirteen patients had an EDSS score lower than or equal to that they had by the time of NTZ start. In total, 40 of the patients had switched or stopped treatment due to the following reasons: 19 due to positive JCV serostatus, 9 due to disease activity, 7 due to adverse effects or complications (one progressive multifocal leukoencephalopathy), 2 due to pregnancy, and 3 due to aHSCT.

Table 2 shows the NEDA-3 status over time. There was a linear decrease in NEDA-3 during the first 5 years of treatment before the curve flattened due to low number of patients remaining on treatment (Figure 1). The overall proportion of patients having NEDA-3 after 1, 3, and 5 years was found to be 74% (49/66), 50% (33/66), and 23% (15/66), respectively.

Table 3 shows differences between those who achieved NEDA-3 after 3 years and those who did not. Lower age at debut, diagnosis, and at first NTZ treatment in the NEDA-3 group are noted, but these differences are not significant. EDSS at first NTZ treatment, however, was significantly lower in those who achieved NEDA-3 compared to those who had EDA. Furthermore, the EDSS score in 2019 was significantly lower in the NEDA-3 group (Table 4). No differences in CSF investigations before diagnosis and attack frequencies in the last 12 months prior to NTZ treatment were found (calculations not shown).

**Table 3:** Differences between patients with EDA and NEDA-3 after 3 years.

	**EDA (*n* = 33)**	**NEDA-3 (*n* = 33)**	***p*-value**
Age at MS-debut (age), mean ± SD	27.6 ± 10.7	26.3 ± 8.7	0.61
Age at diagnosis (years), mean ± SD	29.5 ± 10.4	28.3 ± 8.1	0.59
Age at first natalizumab dose, mean ± SD	36.3 ± 11.0	33.6 ± 10.1	0.31
EDSS at time of diagnosis, mean ± SD	2.3 ± 1.6	2.2 ± 1.6	0.79
EDSS before first natalizumab treatment, mean ± SD	3.7 ± 2.1	2.7 ± 1.6	0.04
Time from diagnosis to first treatment (years), mean ± SD	3.4 ± 3.3	3.6 ± 3.5	0.80
Having received natalizumab as first line treatment, *n* (%)	6 (18%)	5 (15%)	0.74
EDSS in 2019, mean ± SD	4.5 ± 3.0	2.6 ± 1.6	0.02

*EDA, Evidence of Disease Activity; EDSS, Expanded Disability Status Scale; NEDA-3, No Evidence of Disease Activity*.

**Table 4:** Status 2019.

	**Women (*n* = 49)**	**Men (*n* = 17)**	**Total (*n* = 66)**	***p*-value**
Age (years), mean ± SD	40.8 ± 10.6	37.5 ± 10.8	40.0 ± 10.8	0.29
^*^Disease duration (years), mean ± SD	13.0 ± 7.6	11.1 ± 7.0	12.5 ± 7.5	0.36
EDSS, mean ± SD	3.6 ± 2.5	3.3 ± 2.7	3.6 ± 2.5	0.64
Secondary progressive disease, *n* (%)	8 (16)	0	8 (12)	0.08
Still in treatment with natalizumab, *n* (%)	16 (20)	10 (59)	26 (43)	0.06

* *Time from disease debut to 2019*.

## Discussion

It is well-known that there seems to be an increased efficacy of NTZ with longer exposure (10, 11), but the treatment is limited by the PML risk. The documentation of the long-term effectiveness (12) is thus limited. In the present study, we wanted to study success rates, to mirror the results seen in real-life practice, by using an intention-to-treat approach for statistical analysis. The major reason for changing treatment in the present study was not disease activity but JCV status and JCV seroconversion. Fifteen completed ≥5 years of treatment with NEDA-3 (23%).

The efficacy after 2 years with a NEDA-3 of 68% was similar to the findings in other studies. In a recent longitudinal, retrospective single-cohort study from the Czech Republic, including 193 patients, more than 70% of patients achieved NEDA-3 during each year of NTZ treatment after the second year of treatment (11). In a multicenter study from Italy, the 2-year proportion of patients with NEDA-3 in NTZ-treated patients who previously had been non-responders to interferon beta or glatiramer acetate was 67% (12). In an American observational study (STRIVE), nearly 75% exhibited NEDA during year 2 of treatment with NTZ (13).

In our opinion, the effectiveness data in the present study are good and highly pertinent to real-world decisions in clinical practice. Almost one in four who started with NZT adhered to the treatment and showed no disease activity 5 years later. Despite the high discontinuation rate, and a small risk of rebound after discontinuation, it is worth noticing that the overall disease progression in the total cohort, during an average observation time of 5.2 years, was a mean increase in EDSS of only 0.4. Whether a shorter use of NTZ, for instance 2 years, may cause a far longer stabilizing effect in MS can only be speculated since most patients will switch to another effective treatment after discontinuation. However, our data show that the patients with EDA at 3 years were slightly older (not significantly) and had more advanced disease when they started on NTZ compared to those who achieved NEDA-3. Their EDSS in 2019 was almost one point higher than before first treatment with NTZ, while it was unchanged in those who achieved NEDA-3. This may indicate that NEDA-3 status predicts both long-term stability and better prognosis, and that early treatment is associated with this. Our data are too small to draw a firm conclusion, and the difference in EDSS in 2019 between the cohorts of patients with NEDA-3 and EDA at 3 years (2.6 vs. 4.5) could also just reflect a change in treatment practice—having become more liberal in treating earlier and more aggressively over the years. No difference, however, was seen in patients receiving NTZ as first- or second-line treatment. Our data are, however, too small to assess this adequately, and also too small to evaluate a potential effect of previous DMTs.

In general, our long-term data of effectiveness are somewhat better than found in other studies. In the Czech study, the cumulative NEDA-3 rate after 2 years was 46%, and after 3 years, it was 41% (11). In our study, the overall NEDA-3 dropped from 74% from the first year to 68% after the second, and 50% (*n* = 33) of our patients had received NEDA-3 after 3 years of treatment. In the STRIVE interim analyses, 44% attained overall NEDA over 2 years. These were anti-JC virus seronegative patients with early RRMS (12). In comparison with a Scandinavian cohort study (5), our patients were slightly younger, had a lower baseline EDSS, and the NTZ treatment were initiated at a slightly shorter time after the debut of MS. Our patient population started with NTZ infusions 7.9 ± 6.5 years after the first symptom and 5.9 ± 5.8 years after the diagnosis. The patients in the real-world study from Italy started with NTZ 9.3 years after the first symptom (12). No significant difference in achieving NEDA-3 over time between males and females was seen in our study. Only one patient developed antibodies against NTZ. This is lower than expected. Results from the AFFIRM and the SENTINEL studies have shown persistent anti-NTZ antibodies in 6%, and such antibodies have been associated with lower serum concentrations of NTZ and lower drug effect (14).

Safety data from our study and side effects were as expected from other studies. In total, 16 (24%) patients reported side effects. Most were simple headache and fatigue. These numbers correlate well with numbers from the SENTINEL study where 20% reported side effects; the most common was headache (15). Data from the TOP study showed serious adverse events in 829 of 6,148 patients (13.5%). The AFFIRM study reported a prevalence of hypersensitivity reactions <1% and no cases of anaphylactic reactions (15). One patient in our study developed hypersensitivity reaction during infusion of NTZ requiring adrenaline. Another patient developed PML with only minor sequela from that. Other opportunistic infections can occur during NTZ treatment, but must be considered rare (16). Such were not seen in our cohort.

The retrospective nature of the present study, the small sample size, and the short follow-up time in some of the patients limit the scientific weight of it. In a few cases, EDSS had to be calculated retrospectively when missing in the medical recording, and this must also be regarded a limitation. Applying a control group and including cognitive function analysis and brain atrophy measurements (NEDA-4) (17) would certainly have strengthened the study. However, it provides solid real-world data from a complete and well-described MS population (7, 18).

## Conclusion

The use of NTZ is in real-world practice limited by the risk of progressive multifocal leukoencephalopathy. It is effective, however, and it has high tolerability. NTZ should thus remain an important tool in the medical arsenal against MS.

## Data Availability Statement

The raw data supporting the conclusions of this article will be made available by the authors, without undue reservation.

## Ethics Statement

The studies involving human participants were reviewed and approved by Institutional data protection official. Written informed consent from the participants' legal guardian/next of kin was not required to participate in this study in accordance with the national legislation and the institutional requirements.

## Author Contributions

AJ plotted, analyzed and interpreted the data. He wrote the first draft of the manuscript. EB validated the data and helped revising the manuscript. KA designed the study, helped collect, analyse and interpret the data, and revised the manuscript. All authors read and approved the final manuscript.

## Conflict of Interest

EB has received honoraria for giving lectures from Sanofi. KA has received honoraria for giving lectures from Biogen, Allergan, Roche, Teva, and Novartis. He has also served in an advisory board for Biogen Norway, and has enrolled patients in the tysabri observational program (TOP) study. The remaining author declares that the research was conducted in the absence of any commercial or financial relationships that could be construed as a potential conflict of interest.

## Publisher's Note

All claims expressed in this article are solely those of the authors and do not necessarily represent those of their affiliated organizations, or those of the publisher, the editors and the reviewers. Any product that may be evaluated in this article, or claim that may be made by its manufacturer, is not guaranteed or endorsed by the publisher.
